# SeqKit2: A Swiss army knife for sequence and alignment processing

**DOI:** 10.1002/imt2.191

**Published:** 2024-04-05

**Authors:** Wei Shen, Botond Sipos, Liuyang Zhao

**Affiliations:** ^1^ Department of Infectious Diseases, Key Laboratory of Molecular Biology for Infectious Diseases (Ministry of Education), Institute for Viral Hepatitis The Second Affiliated Hospital of Chongqing Medical University Chongqing China; ^2^ European Molecular Biology Laboratory European Bioinformatics Institute Hinxton Cambridgeshire UK

**Keywords:** performance optimization, real‐time analysis, sequence processing, usability, user‐friendly

## Abstract

In the era of ubiquitous high‐throughput sequencing studies, there is a growing need for analysis tools that are not just performant but also comprehensive and user‐friendly enough to cater to both novice and advanced users. This article introduces SeqKit2, the next iteration of the widely used sequence analysis tool SeqKit, featuring expanded functionality, performance optimizations, and support for additional compression methods. Retaining a pragmatic subcommand architecture, SeqKit2 represents substantial enhancement through the inclusion of 19 additional subcommands, expanding its overall repertoire to a total of 38 in eight categories. The new subcommands add functionality such as amplicon processing and robust, error‐tolerant parsing of sequence records. In addition, three subcommands designed for real‐time analysis are added for periodic monitoring of properties of FASTQ and Binary Alignment/Map alignment records and real‐time streaming from multiple sequence files. The performance of SeqKit2 is benchmarked against the old version of SeqKit, Bioawk, Seqtk, and SeqFu tools. SeqKit2 consistently outperforms its predecessor, albeit with marginally higher memory usage, while maintaining competitive runtimes against other tools. With its broad functionality, proven usability, and ongoing development driven by user feedback, we hope that bioinformaticians will find SeqKit2 useful as a “Swiss army knife” of sequence and alignment processing—equally adept at facilitating ad hoc analyses and seamlessly integrating into larger pipelines.

## Highlights

- SeqKit2 expands its capabilities, doubling the number of subcommands from 19 to 38, and adding support for three more compression file formats.
- SeqKit2 outperforms its predecessor and maintains competitive with other tools.
- SeqKit2 improves user‐friendliness with new features, like, autocompletion, progress bars, and enhanced error handling.

## Graphical abstract

SeqKit2 has significantly expanded its capabilities, doubling the number of subcommands from 19 to 38, and adding support for three more compression file formats. This enhances its versatility and efficiency in sequence and alignment processing. Benchmarking results demonstrate that SeqKit2 consistently outperforms its predecessor and remains competitive with other similar tools. In addition, SeqKit2 has notably improved user‐friendliness with the introduction of new features, such as autocompletion for subcommands and command‐line flags and enhanced error‐handling mechanisms.

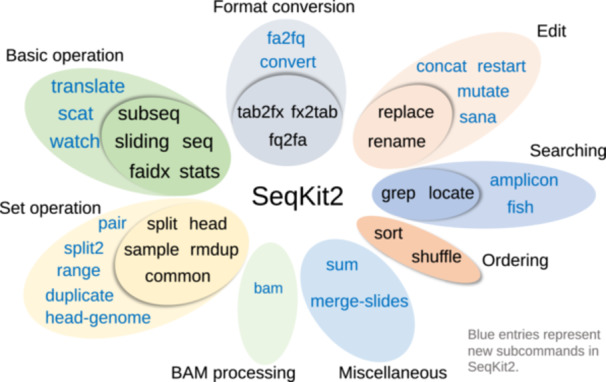

## INTRODUCTION

In the era of high‐throughput sequencing, the volume of raw reads and genomic data, which are mainly stored in FASTA and FASTQ formats in plain or compressed files, significantly increased. This calls for command‐line tools for efficiently processing this data in various scenarios. Some existing utilities, including Bioawk [1], Seqtk [2], BBMaps [3], and SeqKit [4], are widely used by bioinformatic researchers and have wide popularity for years at online question‐and‐answer platforms, such as BioStar [5]. Bioawk extends awk to support biological data formats, including FASTA and FASTQ, providing flexible data processing with built‐in functions. Seqtk has a high processing speed, while it provides a limited number of functionalities. Both BBMaps and SeqKit provide multiple commands for various processing functions, though they have different command structures and command‐line option styles. Lately, a variety of new software options have become available. Pyfastx [6], as a Python package, features fast random access to sequences from both plain and gzip‐compressed FASTA/Q files. SeqFu [7] provides general operations and some specialized utilities. BigSeqKit [8] parallelizes and optimizes commands in SeqKit for processing large‐scale data sets in cluster environments.

SeqKit was originally conceived to deliver efficient and convenient solutions for daily FASTA/Q file manipulations. Following the release of its early version, we continued to optimize its performance, introduce additional features, and enhance usability. Here, we present SeqKit2, which is faster, more user‐friendly, and with expanded functionality.

## RESULTS

**Figure 1:**
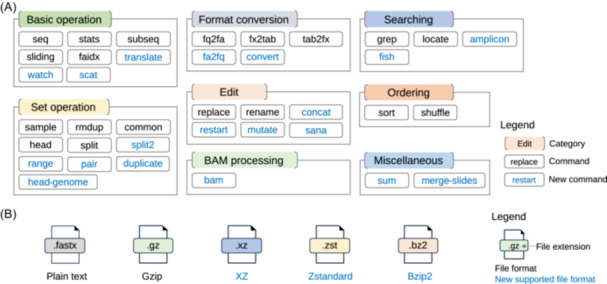
Overview of subcommands and supported file formats in SeqKit2. (A) SeqKit2 subcommands in eight categories, with blue texts representing new ones. (B) Supported compression formats in SeqKit2.

SeqKit2 retains the command‐subcommand architecture from its previous version, allowing users to easily locate specific commands and providing developers with a scalable framework for extending functionality. Nineteen subcommands are added (Figure 1A) and three compression file formats are newly supported (Figure 1B), meanwhile, existing ones are extended with new features. Furthermore, the FASTA/Q files reading and writing speed are significantly improved along with usability.

SeqKit2 comprises 38 subcommands (Figure 1 and Table S1) of eight categories, which doubled since its initial publication 7 years ago. Nineteen new subcommands are added to fulfill users' analysis requirements. For example, “sana” is used for discarding malformed FASTQ records caused by data transfer. Subcommand “amplicon” can be used for extracting amplicon or flanking sequences next to primers in amplicon sequencing data [9]. Subcommand “range” can extract consecutive sequence records in any position of large files. Concatenating sequences with the same identifiers from multiple sequences is a frequently asked operation by beginners at BioStar, which can be easily accomplished by “concat”. Subcommand “sum” is a md5sum‐like tool for digest calculation, which is practical in checking whether two FASTQ files contain identical yet shuffled reads and if multiple circular assemblies from different samples have the same genome with different start positions.

Three subcommands designed for real‐time analysis during sequencing are added. In time‐critical analysis pipelines, it is useful to monitor certain properties of the data as it is being generated. The “watch” subcommand allows the periodical monitoring of various read properties in a FASTQ stream, such as read length, mean quality value, and GC content. The statistics gathered so far are periodically displayed on the terminal as a histogram, saved to PDF files as a histogram, or saved to a TSV file. Similar to the “watch” subcommand, the “bam” subcommand provides online monitoring capability to various Binary Alignment/Map (BAM) record properties, such as mapping quality, alignment accuracy, read length, aligned read length, and many more. We hope that “seqkit bam”, in the context of real‐time analysis pipelines, will complement the established BAM processing tools, such as Samtools [10]. Finally, the “scat” subcommand makes the real‐time processing of FASTQ/FASTA files easier by monitoring a directory structure for new files with a given extension, parsing them in parallel as they are written, merging the individual record streams into one FASTQ output stream which can be easily passed to downstream analysis tools for further analysis.

Existing commands are also extended in functionality. For example, “grep” and “locate” now support searching linear or circular sequences on both strands, with mismatches allowed. Subcommand “faidx”, similar to “samtools faidx”, supports outputting the full header and extracting reverse complement sequences and accepts multiple sequence regions from a file. Subcommand “replace” adds new replacement strings for the record number and corresponding values for matched keys. The subcommand “common” can now detect embedded sequences rather than exactly matched ones.

In addition to introducing new functionalities, SeqKit2 expands its support to three compression formats (Figure 1B), including XZ, Zstandard, and Bzip2. XZ and Zstandard are widely used for storing redundant genomes like SARS‐CoV‐2 for their high compression rate. SeqKit2 seamlessly reads FASTA/Q in different compression formats and writes in one format according to the output file extension.

**Figure 2:**
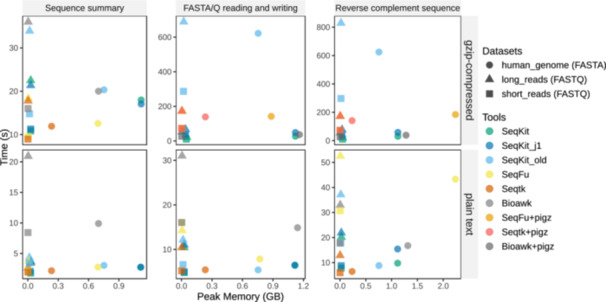
Benchmark results of three common tasks with Bioawk (commit fd40150), Seqtk v1.4, SeqFu v1.20.0, SeqKit v0.3.1.1, and SeqKit v2.8.0. Bioawk, Seqfu, and Seqtk use single‐thread while SeqKit runs with four threads by default. SeqKit_j1 refers to SeqKit v2.8.0 running with one thread. SeqKit_old refers to SeqKit v0.3.1.1. Bioawk+pigz, SeqFu+pigz, and Seqtk+pigz mean the results were piped to pigz v2.4 for compression because these tools inherently generate output in plain text format. GB, gigabytes; s, second.

In terms of performance, SeqKit2 is compared with the first published version, Bioawk, SeqFu, and Seqtk in three common tests, with three data sets in both plain and gzip‐compressed formats (see Methods section). BigSeqKit was not compared because we failed to install it in our cluster. The results (Figure 2 and Table S2) show that, in all tests, SeqKit2 was faster than the old version while consuming more memory. On plain text input files, Seqtk is overall the fastest one, except for short‐read FASTQ file reading and writing, where SeqKit2 is slightly faster. For sequence summary of gzip‐compressed files, Seqtk has the lowest time on the three data sets, followed by SeqFu, SeqKit2, and Bioawk. While for sequence reading and writing and reverse complement sequence computation on gzip‐compressed files, SeqKit2 demonstrated the highest speed, followed by Seqtk+pigz, Bioawk+pigz, and Seqfu+pigz.

The usability of SeqKit2 is also improved. SeqKit2 is actively maintained, and new versions are regularly released with semantic version numbers, which clearly show compatibility changes and therefore ensure the reproducibility of analysis. We also add automatic completion of subcommands and command‐line flags for Bash, Zsh, and Fish in UNIX‐like operating systems and PowerShell in Windows, which significantly improve the user‐friendliness and typing speed when using SeqKit2. Besides, a progress bar is shown when processing two or more files with subcommands, including “stats” and “sum”, which is intuitive in manipulating a long list of large files. Furthermore, various measures have been implemented to enhance the usability of SeqKit2. For instance, a global option “– infile‐list” has been introduced, allowing users to provide a list of input files, thereby overcoming the parameter length limitation imposed by shells. Empty files are allowed and omitted without reporting errors, which makes pipelines using SeqKit2 more robust. To help beginners reduce inappropriate use of input data and command‐line flags, SeqKit2 checks potential misusing and shows warning messages. For example, beginners might use sequence identifiers with a prefix of “>” or “@” for extracting records with the subcommand “grep”. If it happens, SeqKit2 warns that these characters are not a part of sequence identifiers.

## DISCUSSION

Sustained software development is critical for the accuracy of bioinformatic tools [11]. User feedback, including bug reports and feature suggestions, promotes the continuous maintenance and updates of SeqKit2. Since the first publication of SeqKit, the number of subcommands has doubled, increasing from 19 to 38. SeqKit2 goes beyond being a toolkit with a collection of independent tools; most of its subcommands support standard input and output, enabling users to pipe them for advanced processing. Additionally, SeqKit2 demonstrates competitive performance in terms of processing speed, with the highest speed in tasks involving gzip‐compressed FASTA/Q file reading and writing.

SeqKit2 is designed with user‐friendliness in mind, facilitating the rapid processing of sequence data, which proves especially advantageous for beginners. Despite these strengths, SeqKit2 still has room for improvement. For example, it lacks support for random access of FASTQ files and compressed FASTA/Q files, limiting its effectiveness in specific scenarios, such as sorting FASTQ files.

## CONCLUSION

SeqKit2 introduced 19 new subcommands and expanded its support for three additional compression file formats. Concurrently, existing subcommands were enhanced with new features. Furthermore, notable strides were made in both processing speed and user‐friendliness. These advancements solidify SeqKit2 as one of the top choices for sequence data processing.

## METHODS

The parsing performance of SeqKit2 on FASTA/Q records was improved through the optimization of sequence name parsing and the efficient reuse of record objects, meanwhile, compatibility for multiline FASTA/Q formats has been maintained. Subcommands involving a large number of files and Central Processing Unit (CPU)‐intense inexact sequences matching, including “grep”, “locate”, and “amplicon”, were parallelized to utilize the multiple cores of modern CPUs. For subcommands using the hash map for storing a large number of identifiers or sequence data, keys in the string were replaced by their hash values computed by xxhash (https://github.com/cespare/xxhash) for reducing memory occupation. The Gzip and Zstd compression formats supports were provided by http://github.com/klauspost/compress; and the Bzip2 and XZ were provided by https://github.com/dsnet/compress and https://github.com/ulikunitz/xz, respectively. BAM file parsing and Smith–Waterman alignment were provided by bíogo [12]. The plotting of online histograms in the terminal for the “watch” and “bam” subcommands was provided by https://github.com/botond-sipos/thist.

Bioawk (commit fd40150), Seqtk v1.4, SeqFu v1.20.0, SeqKit v0.3.1.1, and SeqKit v2.8.0 were used in performance benchmarking. Three common tasks were performed on a human genome, a long‐read, and a short‐read data set, respectively, both in plain text and gzip‐compressed files. All tests were repeated three times on a single cluster node running with Intel Xeon Gold 6336Y CPU @ 2.40 GHz with 500 GB of RAM running, and average time and memory usage were recorded.

## AUTHOR CONTRIBUTIONS

Wei Shen and Botond Sipos developed SeqKit2. Wei Shen, Botond Sipos, and Liuyang Zhao analyzed the data and interpreted the results. Wei Shen and Botond Sipos wrote the manuscript. All authors have read the final manuscript and approved it for publication.

## CONFLICT OF INTEREST STATEMENT

Botond Sipos is a shareholder of Oxford Nanopore Technologies. The remaining authors declare no conflict of interest.

## ETHICS STATEMENT

No animals or humans were involved in this study.

## Supporting information


**Table S1:** List of SeqKit2 subcommands, with blue texts representing new ones.
**Table S2:** Benchmark results of three common tasks with Bioawk (commit fd40150), Seqtk v1.4, SeqFu v1.20.0, SeqKit v0.3.1.1, and SeqKit v2.8.0.

## Data Availability

SeqKit2 source code and prebuilt executable binary files are available at https://github.com/shenwei356/seqkit. The benchmarking scripts, code, and data to generate the plots are available at: https://github.com/shenwei356/seqkit2_benchmark (v0.2.0). Supplementary information (tables, graphical abstract, slides, videos, Chinese translated version, and update materials) is available online DOI or http://www.imeta.science/.
